# Dietary Intervention during 9 Months with a Hypocaloric Diet, Interaction of the Genetic Variant of Adiponectin Gene rs822393 with Metabolic Parameters

**DOI:** 10.1155/2022/7058389

**Published:** 2022-01-25

**Authors:** Olatz Izaola, David Primo, Daniel de Luis

**Affiliations:** Center of Investigation of Endocrinology and Nutrition, Medicine School and Dept. of Endocrinology and Investigation. Hospital Clinico Universitario, University of Valladolid, Valladolid, Spain

## Abstract

**Background and Aims:**

rs822393 (-4522C/T) genetic variant is associated with hypoadiponectinemia and other metabolic parameters. The aim of our investigation was to analyze the effects of a hypocaloric diet with Mediterranean dietary pattern during 9 months according to genetic variant rs822393 of *ADIPOQ* gene.

**Methods and Results:**

A sample of 269 obese patients was enrolled. Anthropometric and serum parameters (lipid profile, insulin, homeostasis model assessment (HOMA-IR), glucose, C reactive protein, and adipokines) were determined, at basal time and after 3 and 9 months. All patients were genotyped in the rs822393. The genotype distribution was as follow; 176 patients (65.4%) CC, 83 patients CT (30.9%), and 10 patients TT (3.7%). After dietary intervention, the following parameters improved in non-T allele carriers; BMI, weight, fat mass, waist circumference, systolic blood pressure, insulin levels, HOMA-IR, leptin, total cholesterol, and LDL-cholesterol improved significantly. HDL-cholesterol (delta: 5.7 ± 1.1 mg/dl vs. 1.0 ± 0.8 mg/dl; *p* = 0.01), serum adiponectin (delta: 14.4 ± 2.0 ng/dl vs. 7.1 ± 3.1 ng/dl; *p* = 0.02), and adiponectin/leptin ratio (delta: 0.54 ± 0.1 vs. 0.22 ± 0.09 ng/dl; *p* = 0.03). Basal and postintervention HDL cholesterol, adiponectin levels, and adiponectin/leptin levels were lower in T-allele carriers than non-T Allele carriers.

**Conclusion:**

T allele carriers showed lower levels of HDL-cholesterol, adiponectin, and adiponectin/leptin ratio than non-T allele carriers. A medium-term hypocaloric diet with a Mediterranean partner increased adiponectin levels, ratio adiponectin/leptin, and HDL-cholesterol in non-T allele carriers.

## 1. Introduction

Obesity is a public health problem, with huge epidemic ratios, and it is a cornerstone factor in the development of chronic diseases such as cardiovascular events, cancer, diabetes mellitus type 2, hypertension, and dyslipidemia [1]. In all these obesity-related entities, fat mass has an important role with the production of molecules called adipocytokines [2]. In this sense, the adipose tissue participates actively in energy homeostasis, through the secretion of adipokines. Obesity leads to dysregulation of the function of adipokines, which produces various metabolic alterations [2]. Adiponectin is the most abundant circulating adipokine, and levels of adiponectin are reduced in obese subjects and patients with cardiovascular events [3]. Adiponectin has shown the following positive functions in the homeostasis of the organism: enhances energy consumption and fatty acid oxidation, improves overall insulin sensitivity, shows anti-inflammatory and antioxidant properties, as well as cardiovascular protection action [3].

Adiponectin is encoded by adipocyte, C1q, and collagen domain-containing (*ADIPOQ*) gene. This gene is located on chromosome 3 at q27, and it is composed of three exons [4]. Adiponectin shows a strong genetic component, with heritability estimated between 30% and 50% [5]. Only a small number of all known *ADIPOQ* genetic variants have been investigated their metabolic effects after dietary interventions. In addition, contrary of most adipokines, adiponectin levels are decreased in obese subjects and increased after weight reduction [6]. One common genetic variant rs822393 (-4522C/T) is located in the proximal promoter region of the *ADIPOQ* gene. This SNV rs822393 regulates adiponectin promoter activity, and it is related with low circulating levels of adiponectin and insulin sensitivity [7, 8]. In addition, this SNV has been associated with an increase in the risk of diabetes mellitus [9].

In obese subjects, the first therapeutic option is a hypocaloric diet with the aim of improving cardiovascular risk factors [10]. One of the dietary patterns with the greatest beneficial effect on biochemical parameters after weight loss is the Mediterranean diet pattern [11, 12]. The Mediterranean dietary pattern has demonstrated multiple cardiometabolic improvements such as lipid control and glucose improvement [13].

The aim of our study was to analyze the effects of a hypocaloric diet with Mediterranean dietary pattern during 9 months according to genetic variant rs822393 of *ADIPOQ*.

## 2. Subjects and Methods

### 2.1. Subjects and Clinical Investigation

**Figure 1:**
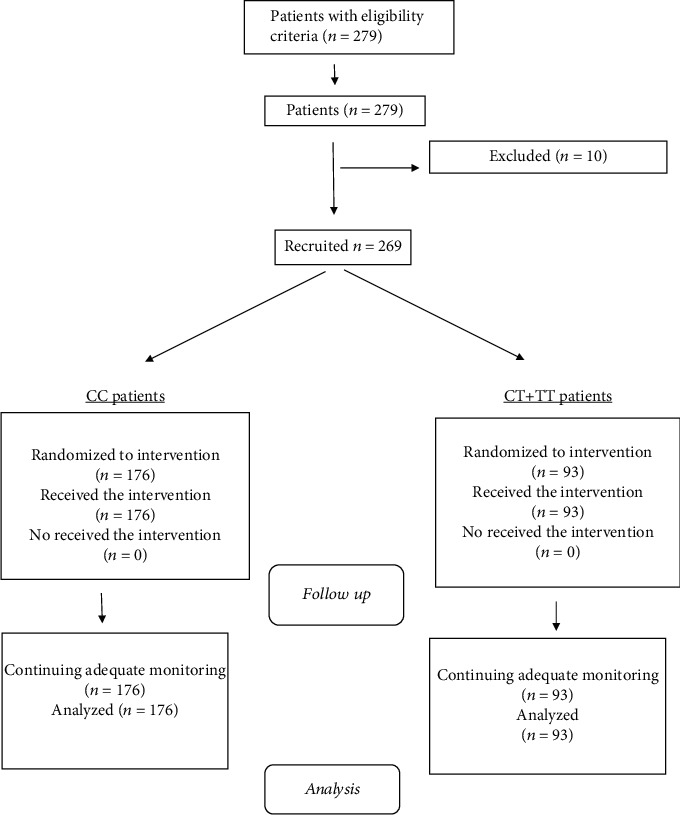
Flow chart of patients.

We designed a nonrandomized interventional study. The population included 269 Caucasian obese patients of a tertiary hospital in an urban area of Spain. We used a continuous consecutive methodology to enroll the subjects. The Institutional Ethics Committee (HCUVA Committee PI8/2017) approved the study protocol. All participants gave written informed consent, and the protocol was performed in accordance with the Helsinki Declaration. Inclusion criteria were the following; body mass index (BMI) ≥ 30 kg/m^2^ and an age over 18 years. Subjects with any of the following criteria were excluded; evidence of any history of cardiovascular or cerebrovascular disease, severe renal or hepatic disorders, alcohol consumption (>20 g/day), active malignant tumor, diabetes mellitus, and receiving medications known to influence lipid levels (fibrates, statins, hormonal therapy, glucocorticoids, and anti-inflammatory drugs) or glucose levels (sulfonylureas, thiazolidinedione, insulin, glucagon-like peptide (GLP-1) receptor antagonists, S-GLT2 (type 2 sodium-glucose cotransporter), DPP-IV (Dipeptidyl peptidase-4) inhibitors, and metformin). Flow chart reported (Figure 1) the 269 recruited patients and 10 excluded patients.

Data of the population were registered at the beginning, after 3 and 9 months of dietary intervention. Adiposity parameters (weight, height, body mass index (BMI), total fat mass, fat mass as % of total body weight, and waist circumference) were reported. We collected blood samples after a 10-hour overnight fasting state. The following parameters were measured; lipid profile (total cholesterol, LDL-cholesterol, HDL-cholesterol, and triglycerides), C-reactive protein (CRP), fasting glucose, insulin, homeostasis model assessment (HOMA-IR), and adipokine levels (leptin, total adiponectin, and resistin). Ratio adiponectin/leptin was calculated.

### 2.2. Dietary Intervention

All 269 subjects received a hypocaloric diet with Mediterranean pattern during a period of 9 months. Dietary intervention consisted of a diet of 1093 cal/day, 53% carbohydrates (144.3 g/day), 27% fats (32.6 g), and 20% proteins (55.6 g/day). The percentage of dietary fats was 65% of monounsaturated fats, 20% of saturated fats, and 15% of polyunsaturated fats. Food color tables were used to teach patients to follow the Mediterranean diet. The Mediterranean dietary pattern included (legumes, vegetables, poultry, whole grains, fish, fresh fruit, using olive oil, and limit unhealthy fats such as margarines, fatty meats, snacks, and industrial pastries). In order to improve compliance of the diet, dietary intervention was recorded each 7 days with a phone call by the same dietitian. Records of daily dietary intake for 6 days (2 weekend days and 4 daily days of the week) were analyzed with a computer-based data evaluation system (Dietosource ®, Gen, Sw). National composition food tables were used as reference [14]. Recommendations of physical activity for patients were aerobic physical activities at least 3 times each week (45 min each). The exercises recommended by our protocol were running, walking, cycling, and swimming. All patients recorded the exercise activity (minutes) with a self-reported questionnaire.

### 2.3. Genotyping *ADIPOQ* Gene

Genomic DNA was obtained from peripheral blood leukocytes using a standard salting out method by QIAamp ® DNA blood kit. Oligonucleotide primers and probes were designed with the Beacon Designer 5.0 (Premier Biosoft International ®, LA, CA, USA). A 50 *μ*L PCR reaction mixture containing 2 *μ*L of genomic DNA, 10 *μ*L of 10× Buffer Reaction (My Taq™ DNA polymerase, BIOLINE), 1.5 *μ*L each of forward and reverse primers, 0.5 *μ*L of My Taq DNA polymerase (My Taq™ DNA polymerase, BIOLINE), and 34.5 *μ*L of water was used in the reaction. PCR amplification was carried out using primer forward: 5′- ACGTTGGATGAAAGCATGACACGGAGCTTC-3′ and reverse 5′-ACGTTGGATGAACCCTCACCCATGTCAGC-3′ in a 2 *μ*L final volume (Termociclador Life Tecnologies, LA, CA, USA). DNA was denatured at 90°C for 2 min; this was followed by 50 cycles of denaturation at 90°C for 30 s, and annealing at 56.1°C for 60 s. We used as internal standard for RT-PCR (GAPDH) with a forward sequence: GTCTCCTCTGACTTCAA and reverse sequence: ACCACCCTGTTGCTGTA. Hardy Weinberg equilibrium was determined with a statistical test (Chi-square).

### 2.4. Biochemical Determinations

Lipid levels (total cholesterol, HDL-cholesterol, and triglycerides), C-reactive protein (CRP), fasting glucose, and insulin were measured on the same day using an automated analyzer COBAS INTEGRA 400 ® (Roche Diagnostic, Montreal, Canada). LDL cholesterol was calculated using Friedewald formula (LDL cholesterol = total cholesterol − HDL cholesterol − triglycerides/5) [15]. The homeostasis model assessment for insulin resistance (HOMA-IR) was calculated with this formula (glucose × insulin/22.5) [16].

All adipokines levels were analyzed by enzyme immunoassay (ELISA) with the following commercial kits; adiponectin (R&D systems, Inc., Minnesota, USA) with a normal range of 8.65-21.43 *μ*g/ml [17], resistin (Biovendor Laboratory, Inc., Brno, Czech Republic) with a normal range of 4-12 ng/ml [18], and leptin (Diagnostic Systems Laboratories, Inc., Texas, USA) with a normal range of 10-100 ng/ml [19]. Adiponectin/leptin ratio was calculated as a ratio between both values.

### 2.5. Anthropometric Parameters

To determine body mass index (BMI), height and weight were measured with a stadiometer (Omrom, LA, CA, USA) and an electrical scale (Omrom, LA, CA, USA), respectively. BMI was calculated with the formula (body weight (kg) divided by height (m^2^)). Waist circumference (WC) was measured with a flexible nonstretchable measuring tape (Type SECA, SECA. Birmingham, UK). Impedanciometry was used to calculate total fat mass with an accuracy of 5 g [20] (EFG, Akern, It). Blood pressure was measured three times and later it was obtained at the mean using a sphygmomanometer (Omrom, LA, CA., USA).

### 2.6. Statistical Analysis

Sample size was determined to detect differences over 5 mg/dl of HDL-cholesterol after dietary intervention with 90% power and 5% significance (*n* = 265). The Kolmogorov–Smirnov test was used to analyze variable distribution. All analysis was performed under a dominant genetic model with rs822393 T-allele as the risk allele (CC vs. CT + TT). A descriptive analysis of the data (mean, standard deviation, frequency of the genotypes) and subsequently an inferential analysis (*X*^2^ test, Student *t*-test and nonparametric test) were carried out. Bonferroni test was applied for multiple testing to reduce type I error in association analysis. The statistical analysis to evaluate the interaction between the gene and the dietary intervention was performed using ANCOVA (covariance analysis) adjusted by age, sex, and BMI modeling the dependent variable with the starting values. These statistical analyses were performed using IBM SPSS version 23.0 software package (SPSS Inc. Chicago, IL). Significance was assumed for *p* < 0.05.

## 3. Results

The genotype percentages were the following; 176 patients (65.4%) CC, 83 patients CT (30.9%), and 10 patients TT (3.7%). The allelic frequencies were C (0.82) and T (0.18). This genetic variant of *ADIPOQ* gene was in Hardy Weinberg equilibrium (*p* = 0.38).

The mean age of the 269 obese Caucasian subjects was 44.1 ± 3.9 years (range: 21-53). The mean body mass index (BMI) was 35.3 ± 1.8 kg/m^2^ (range: 30.4-36.1). Gender distribution was the next; 199 females (74.0%) and 70 males (26.0%). Gender distribution was similar in the three genotype groups (CC; 25.6% males vs. 74.4% females, CT; 26.5% males vs. 73.5% females and TT; 30.0% males vs. 70.0% females: *p* = 0.57). Besides, the mean age was similar in these three genotype groups (CC; 43.9 ± 1.1 years vs. CT; 44.2 ± 2.1 years vs. TT; 44.0 ± 1.3 years: *p* = 0.27). Since the patients with TT genotype presented a low frequency, the analysis was carried out with a dominant genetic model with rs822393 T-allele as the risk allele (CC vs. CT + TT).

Patients reached the dietary recommendations as prescribed in material and method section, total caloric amount of 1006.1 ± 108.9 calories. The percentages of macronutrients were 53.3% from carbohydrates, 20.0% from proteins, and 26.7% from lipids. Percentages of fats were the following; 65.4% from monounsaturated fats, 20.2% from saturated fats, and 14.4% from polyunsaturated fats, without statistical differences between genotype groups (data not shown). Basal physical activity of aerobic exercises was similar in both genotype groups (CC vs. CT + TT) (113.3 ± 22.8 min/week vs. 111.9 ± 21.9 min/week; *p* = 0.53), too. In addition, after 9 months of the study, this physical activity was similar (114.9 ± 14.1 min/week vs. 113.2 ± 12.8 min/week; *p* = 0.48).

### 3.1. Adiposity Parameters and Blood Pressure

Table 1 shows adiposity and clinical parameters before and after the hypocaloric diet with Mediterranean pattern. At the basal time, no differences were observed in anthropometric parameters and blood pressure values. All these parameters were significantly improved after dietary intervention in both genotype groups (*p* < 0.05 compared to baseline from 3 and 9 months) (Table 1). After this specific caloric restriction and in both genotype groups at 9 months (CC vs. CT + TT), BMI (delta: −2.3 ± 0.1 kg/m^2^ vs. −2.1 ± 0.2 kg/m^2^; *p* = 0.19), weight (delta: −5.9 ± 1.1 kg vs. −6.1 ± 1.9 kg; *p* = 0.23), fat mass (delta: −4.8 ± 1.2 kg vs. −4.9 ± 1.1 kg; *p* = 0.41), waist circumference (delta: −5.4 ± 0.5 cm vs. −6.6 ± 0.3 cm; *p* = 0.42), and systolic blood pressure (delta: −5.1 ± 2.1 mmHg vs. −5.8 ± 1.9 mmHg; *p* = 0.32) decreased. No differences between the improvement of both genotype groups were detected. Diastolic blood pressure remained unchanged.

### 3.2. Biochemical Assays

For rs822393 T- allele as the risk allele, we did not observe differences in basal biochemical parameters with a dominant model (CC vs. CT + TT) (Table 2). Only HDL levels were lower in T allele carriers than non-T allele carriers. Insulin, HOMA-IR, total cholesterol, and LDL cholesterol levels improved in both genotype groups (*p* < 0.05 compared to baseline from 3 and 9 months). After the diet intervention and in both genotype groups (CC vs. CT + TT at 9 months); insulin levels (delta: −2.6 ± 0.9 UI/L vs. 2.5 ± 0.3 UI/L; *p* = 0.49) and HOMA-IR (delta: −1.7 ± 0.2 units vs. −1.9 ± 0.4 units; *p* = 0.48), total cholesterol (delta: −12.9 ± 4.1 mg/dl vs. −13.0 ± 3.0 mg/dl; *p* = 0.39), and LDL cholesterol (delta: −10.9 ± 3.1 mg/dl vs. −11.4 ± 2.1 mg/dl; *p* = 0.30) improved. These improvements were similar in both genotype groups. Moreover, HDL-cholesterol levels (delta: 5.7 ± 1.1 mg/dl vs. 1.0 ± 0.8 mg/dl; *p* = 0.01) improved only in non-T allele carriers.

### 3.3. Adipokine

Table 3 shows modifications of all adipokines and the ratio adiponectin/leptin. Leptin levels were significantly improved after dietary intervention in both genotype groups (*p* < 0.05 compared to baseline from 3 and 9 months). After dietary intervention and in non-T-allele carriers (CC vs. CT + TT), serum adiponectin (delta at 9 months: 14.4 ± 2.0 ng/dl vs. 7.1 ± 3.1 ng/dl; *p* = 0.02) increased. Finally, adiponectin/leptin ratio improved in non-T allele carriers (delta at 9 months: 0.54 ± 0.1 vs. 0.22 ± 0.09 ng/dl; *p* = 0.03). Basal and postintervention adiponectin levels and adiponectin/leptin ratio were lower in T-allele carriers than non-T allele carriers.

## 4. Discussion

The primary findings of our study were a significant association between the rs822393 polymorphism of *ADIPOQ* gene and the metabolic response after a 9-month a calorie-restricted dietary treatment with a Mediterranean partner. In this obese Caucasian subjects' study, we observed a significant increase of HDL-cholesterol, adiponectin levels, and ratio adiponectin/leptin in non-T allele carriers. Additionally, T allele carriers show throughout the study lower levels of HDL-cholesterol, adiponectin, and adiponectin/leptin ratio than non-T allele carriers.

This genetic variant of the *ADIPOQ* gene presents scarce studies in the literature, and these studies have reported contradictory results. For example, the Healthy Lifestyle in Europe by Nutrition in Adolescents Study [8] has shown a strong association between rs822393 polymorphism, HDL-cholesterol, and APoA1 serum levels. Moreover, in a noninterventional design [21], a lack of association between this single nucleotide variant (SNV) and metabolic parameters was observed. These differences in literature might be explained to the different ethnic groups or even the different ages of the patients (adolescents vs. adults), and finally, the presence of different allele frequencies and penetrance of this SNV in these populations. Moreover, this area of knowledge is of great interest because HDL-cholesterol has a clear relationship with cardiovascular events [22], and rs822393 variant is related with cardiovascular risk score, HDL-cholesterol, and systolic blood pressure [8], too.

A relevant data of our study is the low levels of HDL cholesterol in T allele carriers. As far as we know, this is the first time that it has been described in a Caucasian population; previously Ramya et al. [9] had reported these data in an Indian population. The potential association between this genetic variant and the adiponectin levels would allow us to explain the relationship detected with the HDL-cholesterol levels. The observed low levels of adiponectin could be explained by the fact that rs822393 is an intronic variant with the capacity to modify the alternative-splicing pattern [23]. Therefore, low levels of adiponectin might produce low levels of HDL-cholesterol. From a physiological point of view, adiponectin is capable of modulating the levels of HDL-cholesterol through two mechanisms [24]; first, activation of lipoprotein lipase and ATP-binding cassette transporter A1 and inhibition of hepatic lipase [25]. Second, adiponectin increases the hepatic production of ApoA1, the main apolipoprotein of HDL-cholesterol [23]. To reaffirm our hypotheses, the CARDIA study (coronary artery development in young adults) reported a relationship between this polymorphism and low levels of adiponectin [26], as our data. In these metabolic relationships, insulin could also play a relevant role since an association was shown in other study [7]. In this cross-sectional study, it was reported an association with insulin sensitivity in a Caucasian population, without association with diabetes mellitus [7]. In other studies [9], this SNV conferred a two-fold higher risk towards diabetes mellitus.

In the literature, there is a previous study that has shown an improvement in adiponectin and HDL-cholesterol levels after a caloric restriction of 1500 calories in a short-term period (3 months) [27]. This metabolic response was observed only in non-T allele carriers [27]. Our current intervention design presents a reduction of the caloric intake up to 1000 calories, and the duration of the intervention is higher than previous [27], reaching 9 months. The distribution of macronutrients in both studies was similar. Moreover, the contribution of monounsaturated fats was higher in the present 9-month study (60%) compared to 50% in the previous 3-month study. In the literature, other designs have reported that different genetic variants of the *ADIPOQ* gene were related with biochemical parameters and body weight. For example, rs266729 G allele was associated with higher weight after a 4-year follow-up [11]. In our study, we have not detected a relationship between rs822393 and adiposity parameters. Moreover, one cross-sectional study [7] reported that this SNV was related to body mass index. In this previous study, T allele carriers showed higher BMI than non-T allele carriers.

Finally, the effect on adipokines is not routinely explored in dietary treatment in obese patients, and yet these molecules have broad effects on metabolism [2, 3], particularly adiponectin [28]. It is necessary to consider the determination from a dynamic point of view with other adipokines, for example, the ratio between anti-inflammatory and proinflammatory adipocytokines such as adiponectin/leptin ratio. The adiponectin/leptin ratio is a good marker of fat mass dysfunction and inflammation [29]. For example, an adiponectin/leptin ratio below or near to 0.5 indicates an increase in the metabolic risk [30, 31], as shown by the carriers of the T allele. Moreover, a ratio higher or near than 1 can be considered as normal, as shown by non-T allele carriers.

Some limitations of this research should also be acknowledged when interpreting our findings. The first limitation is that we analyzed one SNV of *ADIPOQ* and other potential SNVs could be implied, too. Second, the lack of a control group without diet might be a bias. Third, the associations between *ADIPOQ* variant and metabolic changes could be modified by other gene-gene or different gene-environmental interactions. Fourth, unfortunately, we did not measure APOA-1 levels or subtypes of HDL particles. Finally, the self-reported dietary intake might include bias of under- or overreporting.

In conclusion, a medium-term hypocaloric diet with a Mediterranean partner increased adiponectin levels, ratio adiponectin/leptin, and HDL-cholesterol, in non-T allele carriers. T allele carriers showed lower adiponectin and HDL cholesterol levels than non-T allele carriers. Further researchers are needed to assess the link between this genetic variant and cardiovascular event in prospective studies of longer duration. With future prospects, these results could be helpful to design clinical strategies, using SNVs of this gene [32] to support the individual treatment of obese patients.

## Figures and Tables

**Table 1:** Anthropometric parameters and blood pressure (mean ± SD).

Parameters	CC (*n* = 176) Male *n* = 45 and female *n* = 131			CT + TT (*n* = 93) Male *n* = 25 and female *n* = 68		
	Basal	3 months	9 months	Basal	3 months	9 months
BMI	35.4 ± 1.1	33.6 ± 1.2^∗^	33.1 ± 1.1^∗^	35.0 ± 1.3	33.2 ± 1.1^∗^	32.9 ± 1.0^∗^
Weight (kg)	91.4 ± 1.2	87.4 ± 2.0^$^	85.5 ± 2.1^$^	90.8 ± 2.1	85.9 ± 2.0^$^	84.7 ± 2.1^$^
Fat mass (kg)	36.8 ± 1.3	33.6 ± 2.0^#^	32.0 ± 1.0^#^	35.9 ± 1.2	32.3 ± 2.0^#^	31.0 ± 1.9^#^
Fat mass as % of total body weight	40.3 ± 0.8	38.4 ± 0.9^#^	37.4 ± 0.5^#^	39.7 ± 0.5	37.6 ± 0.3^#^	36.5 ± 0.4^#^
WC (cm)	112.2 ± 4.3	107.8 ± 3.2^&^	106.8 ± 3.1^&^	110.7 ± 6.0	105.6 ± 4.2^&^	104.1 ± 3.2^&^
SBP (mmHg)	127.1 ± 3.2	123.7 ± 5.1^∗∗^	122.3 ± 4.1^∗∗^	126.9 ± 4.2	122.8 ± 5.1^∗∗^	121.1 ± 4.1^∗∗^
DBP (mmHg)	81.1 ± 3.0	78.7 ± 4.2	79.1 ± 4.1	81.4 ± 3.1	78.8 ± 4.0	78.1 ± 4.3

BMI: body mass index; DBP: diastolic blood pressure; SBP: systolic blood pressure; WC: waist circumference; statistical differences *p* < 0.05, in each genotype group with basal value (∗BMI, ^$^weight, ^#^fat mass and fat mass as % of total body weight, ^&^WC, ∗∗SBP).

**Table 2:** Biochemical parameters (mean ± SD).

Parameters	CC (*n* = 176)			CT + TT (*n* = 93)		
	Basal	3 months	9 months	Basal	3 months	9 months
Glucose (mg/dl)	102.8 ± 8.0	101.1 ± 7.1	99.0 ± 4.1	101.7 ± 6.0	98.9 ± 4.3	98.0 ± 4.2
Total cholesterol (mg/dl)	210.4 ± 4.1	198.6 ± 4.0^$^	197.6 ± 3.1^$^	208.2 ± 4.2	197.1 ± 3.2^$^	195.1 ± 3.1^$^
LDL-cholesterol (mg/dl)	129.9 ± 7.1	121.8 ± 6.2^#^	119.0 ± 4.9^#^	128.5 ± 4.1	119.0 ± 4.2^#^	116.9 ± 3.2^#^
HDL-cholesterol (mg/dl)	52.4 ± 1.6	57.9 ± 1.9^∗^	58.1 ± 1.2^∗^	45.9 ± 2.0^+^	45.5 ± 1.3^+^	46.9 ± 1.5^+^
Triglycerides (mg/dl)	127.2 ± 17.0	119.9 ± 13.2	120.9 ± 9.2	119.1 ± 13.2	110.8 ± 8.1	112.1 ± 7.1
Insulin (mUI/l)	12.1 ± 2.0	9.6 ± 1.2^&^	8.5 ± 1.1^&^	11.3 ± 2.8	8.8 ± 2.2^&^	8.3 ± 2.1^&^
HOMA-IR	3.5 ± 1.1	2.1 ± 1.0^∗∗^	1.8 ± 0.9^∗∗^	3.9 ± 0.9	2.3 ± 1.1^∗∗^	1.9 ± 1.1^∗∗^
CRP	4.2 ± 1.1	4.3 ± 1.3	4.1 ± 1.9	4.3 ± 2.0	4.4 ± 1.9	4.2 ± 1.0

HOMA-IR: homeostasis model assessment; CRP: C-reactive protein. Statistical differences *p* < 0.05, in each genotype group (total cholesterol^$^, LDL cholesterol^#^, HDL cholesterol^∗^,, insulin^&^, HOMA IR^∗∗^). Statistical differences *p* < 0.05, between genotype group (HDL cholesterol between genotypes^+^).

**Table 3:** Serum adipokine levels (mean ± SD).

Parameters	CC (*n* = 176)			CT + TT (*n* = 93)		
	Basal	3 months	9 months	Basal	3 months	9 months
Resistin (ng/dl)	4.9 ± 1.1	4.8 ± 2.0	4.7 ± 1.8	4.1 ± 2.0	4.4 ± 1.9	4.6 ± 1.3
Adiponectin (ug/dl)	29.7 ± 5.1	59.1 ± 4.1^$^	53.1 ± 4.3^$^	14.9 ± 4.0^+^	17.1 ± 4.1^+^	23.1 ± 7.1^+^
Leptin (ng/dl)	78.3 ± 8.6	61.2 ± 4.5^∗^	58.2 ± 4.9^∗^	81.1 ± 8.1	52.8 ± 7.1^∗^	50.0 ± 6.1^∗^
Ratio adiponectin/leptin	0.37 ± 0.2	0.96 ± 0.1#	0.91 ± 0.2#	0.18 ± 0.1^++^	0.32 ± 0.3^++^	0.41 ± 0.3^++^

Statistical differences *p* < 0.05, in each genotype group (^∗^leptin, ^$^adiponectin, ^#^ratio adiponectin/leptin). Statistical differences *p* < 0.05, between genotype group (^+^adiponectin, ^++^adiponectin/leptin ratio between genotypes).

## Data Availability

All data generated or analyzed during this study are included in this article. Further enquiries can be directed to the corresponding author.
